# Comparison of the efficacy and safety of acetaminophen versus NSAIDs for the treatment of chronic pain in older adults with osteoarthritis of the hip and knee: Findings from the randomized, double-blind, parallel-group, non-inferiority RETHINK study

**DOI:** 10.1016/j.ocarto.2026.100850

**Published:** 2026-07-06

**Authors:** Makoto Endo, Tsutomu Kawano, Masami Tokunaga, Shinya Kawahara, Taro Mawatari, Toshihiko Hara, Yasutaka Tashiro, Masahiro Matsuda, Taishi Sato, Shoji Baba, Akihiko Hamasaki, Toshio Takano, Masumi Miyake, Hiroyuki Aono, Sanae Sakamoto, Tempei Miyaji, Mototsugu Shimokawa, Sadamoto Zenda, Yasuharu Nakashima, the RETHINK study group

**Affiliations:** a Department of Orthopaedic Surgery, Kyushu University, Fukuoka, Japan; b Department of Orthopaedic Surgery, Kyushu Rosai Hospital, Kitakyushu, Japan; c Department of Orthopaedic Surgery, Fukuoka Orthopaedic Hospital, Fukuoka, Japan; d Department of Orthopaedic Surgery, Hamanomachi Hospital, Fukuoka, Japan; e Department of Orthopaedic Surgery, Aso Iizuka Hospital, Iizuka, Japan; f Graduate School of Health and Social Services, Saitama Prefectural University, Koshigaya, Saitama, Japan; g Ayumi Pharmaceutical Corporation, Tokyo, Japan; h Fukuoka Data Centre of Clinical Trials, Fukuoka, Japan; i Meaningful Outcome Consulting Inc., Tokyo, Japan; j Department of Biostatistics, Yamaguchi University Graduate School of Medicine, Ube, Japan; k Department of Radiation Oncology, National Cancer Centre Hospital East, Kashiwa, Japan

**Keywords:** Acetaminophen, Non-steroidal anti-inflammatory drugs, Osteoarthritis, pain improvement, Older adults, Clinical trial

## Abstract

**Objective:**

This study aimed to evaluate the non-inferiority of acetaminophen compared with non-steroidal anti-inflammatory drugs (NSAIDs) for the treatment of chronic osteoarthritis-related pain in older adults.

**Design:**

This multicenter, randomized, double-blind, parallel-group study enrolled patients aged 65 years or older with osteoarthritis-related pain. Participants were randomly assigned to receive acetaminophen (1800 mg/day) or NSAIDs (loxoprofen 180 mg/day or celecoxib 200 mg/day). The primary endpoint was the change in Brief Pain Inventory (BPI) item 3 (worst pain) score from baseline to week 8. The secondary endpoints included the change in BPI item 3 score from baseline to week 4, quality of life, gastrointestinal disorders, and renal and liver function parameters.

**Results:**

Of the 400 patients enrolled, 191 and 197 were in the acetaminophen (mean age 73.6 years; 83.2% female) and NSAID groups (mean age 73.3 years; 74.6% female), respectively. The least-squares mean change in BPI item 3 scores at 8 weeks was −1.79 in the acetaminophen group and −1.94 in the NSAIDs group. The between-group difference in BPI item 3 scores change was 0.14 (95% CI, −0.33 to 0.61). No major safety concerns were identified; however, gastrointestinal disorders occurred more frequently with NSAIDs and were the most common cause of treatment discontinuation.

**Conclusions:**

In this RETHINK study, acetaminophen achieved a similar reduction in osteoarthritis-related pain to NSAIDs in older adults after eight weeks; however, non-inferiority was not demonstrated. In terms of adverse events, acetaminophen was associated with fewer gastrointestinal disorders. These findings suggest that treatment choice may depend on the balance between analgesic efficacy and safety considerations.

**Trial registration number:**

The study is registered in the Japan Registry of Clinical Trials (jRCTs071200112).

## 1 Introduction

Osteoarthritis is a significant cause of chronic musculoskeletal pain and a leading cause of physical disability in older adults. The prevalence of osteoarthritis increases with age, reaching about 17% at age 65, with the hip and knee joints being the most commonly affected joints [1]. In many developed countries, including Japan, the number of patients with diseases is expected to increase as the population ages; therefore, the social importance of several diseases has been steadily rising.

The primary therapeutic options for osteoarthritis include analgesics, exercise therapy, orthotic therapy, and surgery [2]. In particular, oral analgesics are essential treatment options for osteoarthritis, and the commonly used oral non-opioid analgesics include acetaminophen; non-selective non-steroidal anti-inflammatory drugs (NSAIDs) such as loxoprofen, ibuprofen, and diclofenac; and selective cyclooxygenase-2 (COX-2) inhibitors such as celecoxib [3]. Several clinical trials have compared the efficacy of these drugs; however, dosage and eligibility criteria varied across these trials. Hence, conclusions regarding the most appropriate drug for pain relief remain controversial [4]. Moreover, the adverse events associated with these drugs are likely to have more pronounced effects in older adult patients due to factors such as age-related pharmacokinetic changes and polypharmacy [5]. Particularly, gastrointestinal complications and cardiovascular disorders are major adverse events associated with the use of these analgesics, with the risk of gastrointestinal complications being higher for NSAIDs and older adults [[6], [7], [8]]. Consequently, analgesics for older adults with osteoarthritis should be selected based on data from prospective comparative studies that specifically assess the efficacy and safety of these drugs in this population; however, only a few high-quality comparative studies have been conducted. Therefore, we conducted a multicenter, randomized, double-blind, parallel-group study, the RETHINK study, to compare the efficacy of acetaminophen and NSAIDs in treating osteoarthritis-related pain in older adults. We hypothesized that acetaminophen is less toxic than NSAIDs and accordingly set the primary endpoint as non-inferiority of the analgesic effects of acetaminophen compared to those of NSAIDs at week 8.

## 2 Methods

### 2.1 Study design

The study design for the RETHINK study has been described previously [9]. This multicenter, randomized, double-blind, parallel-group study examined the efficacy and safety of acetaminophen versus NSAIDs in older adult patients with osteoarthritis-related pain. Potentially eligible patients were informed about the study by their attending physicians, and those who consented were enrolled between May 2021 and June 2023. The study was conducted at five Japanese institutions: Kyushu University Hospital, Fukuoka Orthopaedic Hospital, Aso Iizuka Hospital, Hamanomachi Hospital, and Kyushu Rosai Hospital. The study protocol was approved by the Kyushu University Hospital Certified Institutional Review Board for Clinical Trials (CRB: Certification No. CRB718005) before patient enrolment. The study was conducted in accordance with the Declaration of Helsinki and all applicable regulatory requirements.

### 2.2 Patients

Eligible participants were patients aged ≥65 years with hip and/or knee osteoarthritis diagnosed according to the American College of Rheumatology criteria [10,11]. At enrolment, osteoarthritis-related pain was assessed using the numerical rating scale corresponding to item 3 of the Brief Pain Inventory (BPI) [12]. In this study, pain assessment was limited to pain in the affected hip and/or knee joint(s). Based on previously published cut-off points indicating the transition from mild to moderate pain intensity [13], a score of ≥3 for the worst pain experienced during the previous 24 h was set as an eligibility criterion for this study. In addition, the following laboratory cut-off values were used for patient enrolment: aminotransferase (AST) levels ≤60 U/L, alanine transaminase (ALT) levels ≤84 U/L for men and ≤46 U/L for women, gamma-glutamyl transpeptidase (γ-GTP) levels ≤128 U/L for men and ≤64 U/L for women, total bilirubin (T-Bil) ≤ 30.0 mg/dL, and estimated glomerular filtration rate (eGFR) ≥ 30 mL/min/1.73 m^2^.

The exclusion criteria were as follows: patients scheduled for surgery during the study period; those who had contraindications or were hypersensitive to the study medications; patients who required treatment for pain unrelated to osteoarthritis; patients with inflammatory bowel disease, kidney disease, coagulation disorders, uncontrolled hypertension, psychiatric disorders, or dementia; heavy alcohol consumers; and patients who were undergoing treatment for upper gastrointestinal tract ulcers or reflux esophagitis. In addition, patients who were deemed ineligible by the investigator were excluded from the study.

Patients were required to initiate the study medication within 30 days after providing informed consent and to continue treatment for eight weeks. Before initiation, a medication washout period of at least three days was implemented to minimize the potential influence of prior analgesic treatments.

### 2.3 Interventions

Patients were assigned to receive either acetaminophen (600 mg three times daily) or NSAIDs (loxoprofen [60 mg, three times daily] or celecoxib [100 mg, twice daily], with a placebo administered once daily for eight weeks. The study medication doses were selected based on routine clinical use; in particular, an acetaminophen dose of 1800 mg/day was selected to reflect the commonly reported average daily use in international clinical practice [14]. The patient and the doctor were blinded to the treatment allocation. Randomization and blinding procedures were explained to all patients during the informed consent process, including the possibility of receiving any of the study medications. At each institution, an unblinded pharmacist who was not involved in outcome assessment managed drug allocation and dispensing. Medication compliance was promoted and assessed using patient-completed medication diaries throughout the study period. The study medications were provided by Ayumi Pharmaceutical Corporation in identical forms, making it impossible to visually distinguish between the drug types. The use of analgesics other than the study medications and topical analgesic patches, as well as surgical treatment and prophylactic use of proton pump inhibitors, H2-receptor antagonists, and gastric mucosal protection drugs, was prohibited during the study. In Japan, prophylactic co-prescription of gastroprotective agents is generally not permitted except in patients with a history of recurrent gastrointestinal disease. No rescue medication for pain exacerbation was permitted during the study period.

The patients were randomly assigned to the treatment groups using Fountayn (Fountayn, Inc., Ohio, USA), an Electronic Data Capture system. Pain intensity at enrolment was categorized into two predefined ranges: BPI item 3 scores of 3–6 and 7–10. This categorization, together with study institution and affected joint site (hip or knee), was used as an allocation stratification factor to ensure a balanced distribution of baseline pain severity between the treatment groups. Eligible patients were randomly assigned to receive the medication. Blinding was lifted at the end of the study; however, a participant could be unblinded if a serious adverse event or other incidents occurred. Serious adverse events were reported to the site administrator and investigator and, as appropriate, to the Kyushu University Hospital CRB and the sponsor. No personal information was collected during the reporting process.

### 2.4 Follow-up

Each patient was administered the study medication for eight weeks, starting at week 0. The patients visited the institution at weeks 0, 4, and 8 for medical interviews, blood tests, and urinalyses. At each visit, the patients were required to use electronic patient-reported outcomes to complete questionnaires, including the BPI, the Short Form-8 (SF-8) [15], and Gastrointestinal Symptom Rating Scale (GSRS) [16]. Patient-reported outcomes were self-completed by participants on an electronic tablet. The GSRS assessment at week 2 was conducted remotely using a paper questionnaire, without an on-site visit, owing to the potential for gastrointestinal disorders to occur within a short period. The study protocol specified discontinuation of study treatment if the BPI item 3 score worsened by 4 points or more from baseline during the study period to ensure patient safety.

### 2.5 Outcomes

The primary endpoint was the change from baseline to week 8 in the BPI–Short Form item 3 score, which assesses worst pain intensity. This item is rated on an 11-point numerical rating scale ranging from 0 (“no pain”) to 10 (“pain as bad as you can imagine”), with a 24-h recall period. The exact wording of BPI item 3 is: “Please rate your pain by circling the one number that best describes your pain at its WORST in the past 24 h” [10]. This scale is a widely used self-administered questionnaire that has been validated for assessing chronic pain conditions, including osteoarthritis. Additionally, its Japanese version has been validated [17,18].

The SF-8 is used to measure health-related quality of life (QOL) over the past month, which includes two standardized domains, calculated by weighting each SF-8 item using a norm-based scoring method: the Physical Component Summary (PCS) and the Mental Component Summary (MCS). Higher scores indicate better health-related QOL [19].

The GSRS is a self-administered questionnaire that consists of 15 items used to assess a wide range of symptoms of upper gastrointestinal diseases/disorders. The disorders are rated using a 7-point Likert scale, with responses ranging from “No discomfort at all” to “Very severe discomfort,” and a recall period of “during the past week”. The Japanese version of the scale has been validated [20,21].

The secondary endpoints included change in the BPI item 3 score from baseline to week 4; change in SF-8 score from baseline to weeks 4 and 8; GSRS score obtained at baseline and weeks 2, 4, and 8; changes in eGFR and blood pressure from baseline to weeks 4 and 8, and the proportion of patients with elevated AST, ALT, γ-GTP, ALP, and T-Bil values. In addition, the adverse events were assessed by study physicians at each visit. Patients were also instructed to record symptoms of concern in their medication diaries as supplemental information. The data center was located at the Fukuoka Data Center of Clinical Trials.

### 2.6 Sample size

Previous studies conducted using the Visual Analogue Scale to compare the effects of NSAIDs and acetaminophen expected Visual Analogue Scale score changes of −2 [17,18], with a difference of −1 considered clinically significant [[22], [23], [24], [25], [26], [27]]. Based on these findings, non-inferiority margins in previous studies typically range from approximately 0.8 to 1.0. In the present study, a more conservative non-inferiority margin of 0.6 was prespecified, reflecting a deliberate clinical judgment to avoid overestimating non-inferiority. Sample size calculation using these values, a 2.5% one-sided significance level, and 80% power, resulted in a sample size of 176 per group. Considering a dropout rate of approximately 10% reported in previous trials [28,29], the target number of participants was set at 200 per group, resulting in a total of 400 participants for this study. The primary objective of this study was a non-inferiority comparison of acetaminophen versus NSAIDs as a drug class. Therefore, the sample size calculation was prespecified based on two treatment groups (acetaminophen and pooled NSAIDs), with loxoprofen and celecoxib included as representative NSAIDs commonly used in clinical practice in Japan.

### 2.7 Statistical analysis

Continuous variables in the baseline data are summarized as means and standard deviations, minimum and maximum values, and medians, whereas categorical variables are summarized as counts and percentages. The full analysis set (FAS) excluded ineligible patients, patients not taking the study medication, and patients with no baseline or assessment data. The per-protocol set (PPS) excluded patients who did not follow the study protocol and patients with less than 75% medication compliance. The safety analysis set included all patients who received at least one dose of the study medication. A PPS analysis was also performed to evaluate the primary endpoint, which was the change in BPI item 3 score from baseline to week 8, and to assess the non-inferiority of acetaminophen versus NSAIDs, with a non-inferiority margin of 0.6 and a one-sided significance level of 0.025. Differences between the acetaminophen and NSAIDs groups were evaluated by analyzing the least squares mean (LSM) change and 95% confidence intervals (CIs). Non-inferiority testing was conducted using a mixed-effect model with repeated measures, considering the baseline value, treatment group (acetaminophen group vs NSAIDs group), evaluation timepoint, interaction between treatment group and evaluation timepoint, and allocation adjusting factors as covariates. Missing data were implicitly imputed using the mixed-effects model with repeated measures, assuming that the data were missing at random.

## 3 Results

### 3.1 Baseline characteristics of the patients

**Figure 1:**
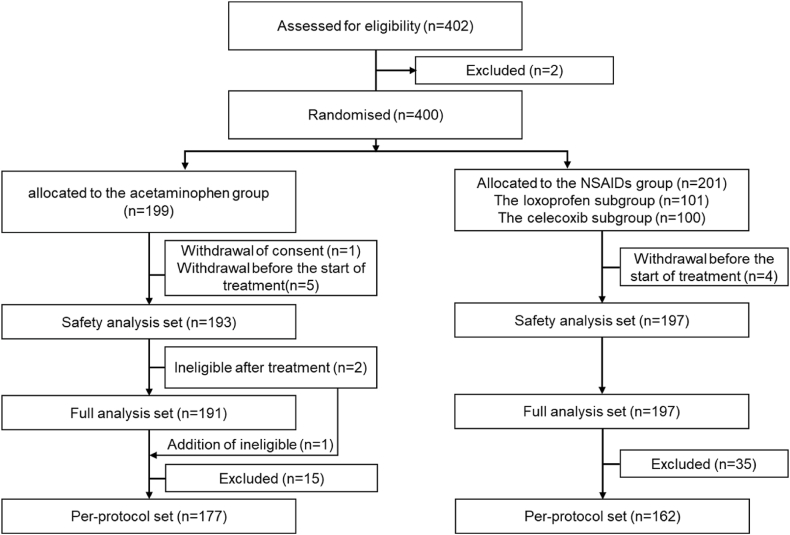
Flowchart of patient selection

Between May 2021 and June 2023, 402 patients were screened, of which 400 were included in the study and randomly assigned to the acetaminophen group (n = 191) and the NSAIDs group (n = 197; 98 in the loxoprofen subgroup and 99 in the celecoxib subgroup). These patients were analyzed as the FAS (Fig. 1). The baseline characteristics of the FAS are summarized in Table 1. The mean ages of the patients in the acetaminophen and NSAID groups were 73.6 and 73.3 years, respectively, and their mean weights were 59.3 and 59.7 kg, respectively. Additionally, 83.2% and 74.6% of the patients in the acetaminophen and NSAIDs groups, respectively, were women. The prevalence of osteoarthritis of the knee was 51.3% in the acetaminophen group and 52.3% in the NSAIDs group. In total, 40.8% and 41.1% of patients in the acetaminophen and NSAIDs groups, respectively, had a BPI item 3 score of ≥7 points. Except for the higher proportion of female patients in the acetaminophen group than in the NSAIDs group, the baseline characteristics of the patients were well balanced between the two groups.

**Table 1:** Baseline characteristics of the patients

Characteristics		Acetaminophen group (N = 191)	NSAIDs group		
			All (N = 197)	Loxoprofen (N = 98)	Celecoxib (N = 99)
Age (years)	Mean ± SD	73.6 ± 5.8	73.3 ± 5.7	72.8 ± 5.9	73.7 ± 5.5
	[min-max]	[65.0–91.0]	[65.0–92.0]	[65.0–92.0]	[65.0–87.0]
	Median	72.0	73.0	72.0	73.0
Weight (kg)	Mean ± SD	59.3 ± 11.4	59.7 ± 11.4	59.8 ± 11.4	59.6 ± 11.5
	[min-max]	[37.3–106.2]	[36.7–101.5]	[36.7–101.5]	[38.1–85.9]
	Median	56.9	58.1	57.7	58.2
BMI	Mean ± SD	24.9 ± 4.2	24.8 ± 3.9	24.8 ± 3.8	24.7 ± 4.1
	[min-max]	[17.7–39.4]	[16.5–36.6]	[16.5–36.6]	[16.5–36.0]
	Median	24.2	24.1	24.2	24.1
Sex, n (%)	male	32 (16.8)	50 (25.4)	24 (24.5)	26 (26.3)
	Female	159 (83.2)	147 (74.6)	74 (75.5)	73 (73.7)
Osteoarthritis, n (%)	hip	93 (48.7)	94 (47.7)	46 (46.9)	48 (48.5)
	Knee	98 (51.3)	103 (52.3)	52 (53.1)	51 (51.5)
BPI item 3 score, n (%)	3∼6	113 (59.2)	116 (58.9)	57 (58.2)	59 (59.6)
	7∼10	78 (40.8)	81 (41.1)	41 (41.8)	40 (40.4)

BMI, body mass index; BPI, brief pain inventory

### 3.2 Efficacy

**Figure 2:**
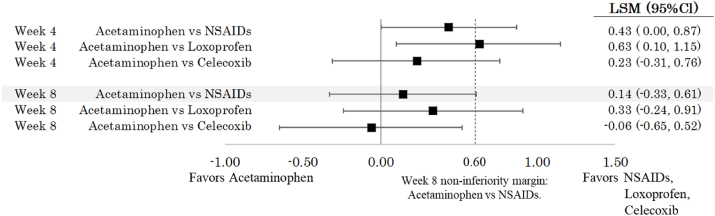
Differences in pain score changes from baseline, measured using the BPI item 3 This figure illustrates the difference in LSM change from baseline between the acetaminophen and the nonsteroidal anti-inflammatory drugs (NSAIDs), including a comparison of the pooled NSAIDs and exploratory comparisons with individual NSAIDs (loxoprofen and celecoxib). The primary non-inferiority analysis was prespecified for the comparison between acetaminophen and pooled NSAIDs at week 8 (highlighted); the non-inferiority margin (0.6) applies only to this primary comparison. The results for week 4 and comparisons involving individual NSAIDs are provided as supplementary information. Squares: LSM; vertical lines: 95% confidence intervals (CIs).

**Figure 3:**
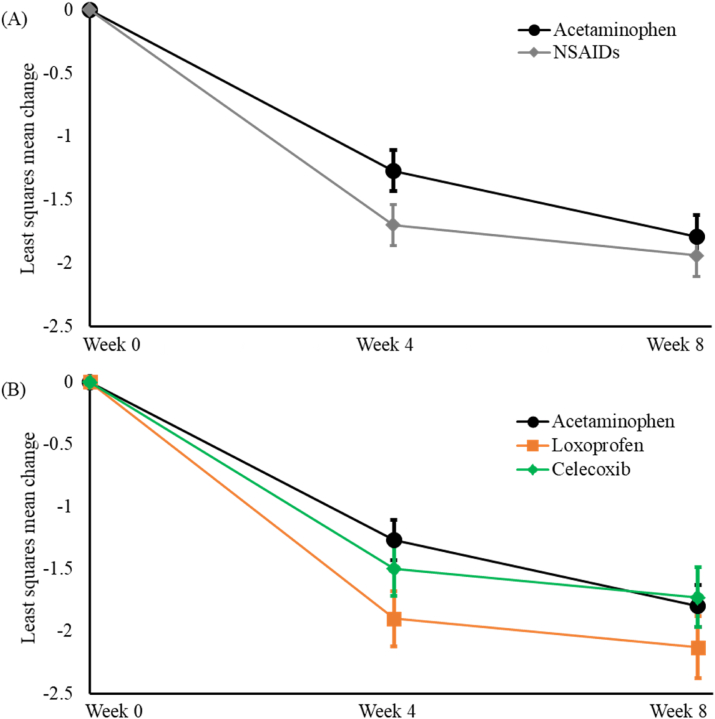
Changes in pain scores from baseline, measured using the BPI item 3 This figure illustrates the LSM change in pain reduction for (A) the acetaminophen and the nonsteroidal anti-inflammatory drugs (NSAIDs), and (B) the acetaminophen, the loxoprofen, and the celecoxib. Regarding the change in BPI item 3 score at 8 weeks, the LSM change was −1.79 (95% CI, −2.12 to −1.46) in the acetaminophen group and −1.94 (−2.27 to −1.61) in the NSAIDs group.

The LSM change in BPI item 3 score (0–10 NRS) at week 8 was −1.79 (95% CI, −2.12 to −1.46) in the acetaminophen group and −1.94 (95% CI, −2.27 to −1.61) in the NSAIDs group. The difference in LSM change between the two groups was 0.14 (95% CI, −0.33 to 0.61). As the upper limit of the 95% CI exceeded the non-inferiority margin of 0.60, statistical non-inferiority was not demonstrated (Fig. 2). The change in BPI item 3 score at 4 weeks was −1.27 (95% CI, −1.58 to −0.96) in the acetaminophen group and −1.70 (95% CI, −2.01 to −1.39) in the NSAIDs group. The difference in the BPI item 3 score change between the two groups was 0.43 (95% CI, 0.00 to 0.87) (Fig. 3).

The PPS included 177 patients in the acetaminophen group and 162 in the NSAIDs group. At week 8, the LSM change in BPI item 3 score (0–10 NRS) was −1.86 (95% CI, −2.19 to −1.53) in the acetaminophen group and −2.07 (95% CI, −2.19 to −1.72) in the NSAIDs group, with an LSM difference between the two groups of 0.20 (95% CI, −0.28 to 0.69). Evaluation of the NSAIDs subgroups showed that the change in BPI item 3 score at 8 weeks was −2.13 (95% CI, −2.60 to −1.66) in the loxoprofen subgroup and −1.73 (95% CI, −2.22 to −1.24) in the celecoxib subgroup. The difference in the score change compared to the acetaminophen group was 0.33 (95% CI, −0.24 to 0.91) for the loxoprofen subgroup and −0.06 (95% CI, −0.65 to 0.52) for the celecoxib subgroup (Fig. 3).

Exploratory subgroup analyses suggested a trend of greater efficacy of acetaminophen in female patients and in those with higher body weight and elevated body mass index (Supplementary Fig. S1) compared to their counterparts.

The change in MCS of the SF-8 scores at 8 weeks was 1.22 (95% CI, 0.29 to 2.15) in the acetaminophen group and 2.02 (95% CI, 1.07 to 2.97) in the NSAIDs group, with a difference of −0.80 (95% CI, −2.12 to 0.53) between the two groups. The change in PCS at 8 weeks was 2.41 (95% CI, 1.36 to 3.47) in the acetaminophen group and 3.86 (95% CI, 2.78 to 4.93) in the NSAIDs group, with a difference of −1.44 (95% CI, −2.94 to 0.06) between the two groups (Supplementary Fig. S2).

### 3.3 Safety

Gastrointestinal disorders, and renal dysfunction occurred in 33 (17.1%) and two (1.0%) patients in the acetaminophen group and 51 (25.9%) and four (2.0%) patients in the NSAIDs group (Supplemental Table S1), respectively. A hepatobiliary adverse event (grade 1 hepatobiliary disorder) was observed in one patient in the acetaminophen group.

The treatment protocol was discontinued owing to adverse events in 6 of 193 patients (3.1%) in the acetaminophen group and in 18 of 197 patients (9.1%) in the NSAIDs group (Table 2). The most common adverse event leading to discontinuation of protocol treatment was gastrointestinal disorders, in one (0.5%) patient in the acetaminophen group and 14 (7.1%) patients in the NSAIDs group. In the NSAIDs group, eight (8.2%) of the 98 patients treated with loxoprofen and six (6.1%) of the 99 patients treated with celecoxib discontinued the treatment because of gastrointestinal disorders.

**Table 2:** Incidence of patient dropout due to adverse events

Adverse event category	Acetaminophen	Loxoprofen	Celecoxib
	(n = 193)	(n = 98)	(n = 99)
All	6 (3.1)	10 (10.2)	8 (8.1)
Gastrointestinal disorders	1 (0.5)	8 (8.2)	6 (6.1)
Infections and infestations	1 (0.5)	0 (0.0)	0 (0.0)
Injury, poisoning, and procedural complications	1 (0.5)	0 (0.0)	0 (0.0)
Investigations	0 (0.0)	1 (1.0)	0 (0.0)
Metabolism and nutrition disorders	0 (0.0)	0 (0.0)	1 (1.0)
Respiratory, thoracic, and mediastinal disorders	0 (0.0)	1 (1.0)	0 (0.0)
Skin and subcutaneous tissue disorders	3 (1.6)	0 (0.0)	1 (1.0)

**Figure 4:**
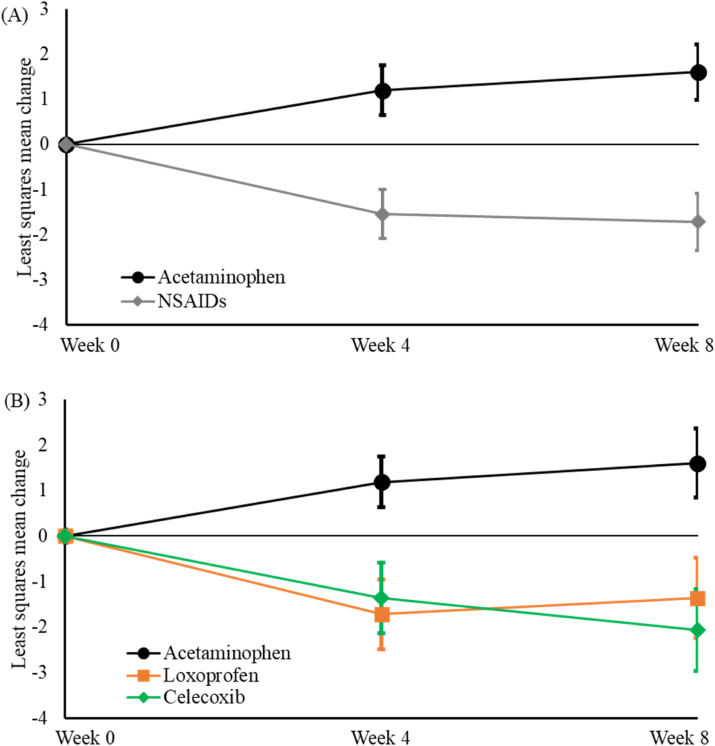
Changes in eGFRs from baseline This figure illustrates the LSM change in (A) the acetaminophen and the nonsteroidal anti-inflammatory drugs (NSAIDs), and (B) the acetaminophen, the loxoprofen, and the celecoxib.

The mean GSRS score increased by 0.01 and 0.08 in the acetaminophen and NSAIDs groups, respectively, at the two-week timepoint, with a particularly notable increase observed in the loxoprofen subgroup (Supplementary Fig. S3). After week two, the scores remained consistent and did not exhibit a clear upward trend. The change in eGFR at 8 weeks (mL/min/1.73 m^2^) was 1.60 (95%CI, 0.40 to 2.80) in the acetaminophen group and −1.71 (95% CI, −2.95 to −0.48) in the NSAIDs group, with a difference of 3.31 (95% CI, 1.58 to 5.03) between the groups. The loxoprofen and celecoxib subgroups showed a similar decrease in eGFR (Fig. 4). The change in systolic blood pressure values at 8 weeks (mmHg) was 1.21 (95% CI, −0.93 to 3.35) in the acetaminophen group and 4.29 (95% CI, 2.10 to 6.48) in the NSAID group, with a difference of −3.08 (95% CI, −6.14 to −0.02) between the groups (Supplementary Fig. S4). At 8 weeks, 12 of 178 (6.7%) patients in the acetaminophen group and 9 of 167 (5.4%) patients in the NSAID group had AST levels exceeding the upper limit of normal. Similarly, 27 of 178 (15.2%) patients in the acetaminophen group and 7 of 167 (4.2%) patients in the NSAIDs group showed alanine transaminase values higher than the upper limit of normal (Supplementary Table S2).

## 4 Discussion

Analgesics are widely used for the management of osteoarthritis; however, particularly in older adults, treatment should be selected to achieve an appropriate balance between analgesic efficacy and the risk of adverse events. Against this clinical background, we conducted a randomized, double-blind clinical trial to compare the efficacy and safety of acetaminophen with those of NSAIDs in patients aged 65 years or older. To the best of our knowledge, this is the first study to provide comparative evidence on the efficacy and safety of acetaminophen versus NSAIDs for the management of osteoarthritis-related pain, specifically in older adults. Importantly, this study generated high-quality data on the use of these commonly prescribed analgesics in a large cohort of patients aged 65 years or older. These findings may help clinicians select appropriate pharmacological treatment for older patients with osteoarthritis by considering analgesic efficacy, quality-of-life effects, and the risk of adverse events, including gastrointestinal, renal, hepatic, and cardiovascular disorders.

In the present study, acetaminophen at a dose of 1800 mg/day did not demonstrate non-inferiority to NSAIDs in the treatment of osteoarthritis-related pain in older adults over an 8-week period, as the upper limit of the 95% confidence interval for the mean difference in BPI item 3 score was 0.61, slightly exceeding the prespecified non-inferiority margin of 0.6. Thus, the primary endpoint of the study was not met. However, the non-inferiority margin of 0.6 used in this study was more conservative than those adopted in previous trials, reflecting a deliberate clinical judgment to avoid overestimating non-inferiority [30].

Among the study cohort, 100 of the 200 patients in the NSAID group received loxoprofen, a traditional NSAID commonly used in Japan, and 100 received celecoxib, a selective COX-2 inhibitor. The results of the subgroup analyses suggested differences in analgesic efficacy and adverse events between loxoprofen and celecoxib. Specifically, the change in pain scores at 8 weeks showed that celecoxib was less analgesic than loxoprofen and similar to acetaminophen. This finding may, at least in part, be attributable to the fact that acetaminophen was not administered at the maximum approved dose in this study.

In terms of clinical importance, the minimal clinically important difference (MCID) for osteoarthritis-related pain [31], measured with NRS, has been reported to be 0.9 [32]. In the present study, reductions at week 8 exceeded the MCID in both treatment groups, with changes of −1.79 in the acetaminophen group and −1.94 in the NSAIDs group; −2.13 in the loxoprofen group and −1.73 in the celecoxib group. Reductions at week 4 also exceeded the MCID in both treatment groups.

In this study, both groups showed increased changes in MCS and PCS scores at 8 weeks, as measured by the SF-8. The increase in the PCS score exceeded the MCID of 2 points, whereas the change in the MCS score did not reach the MCID of 3 points or higher in either group [15]. These results are consistent with the changes in the PCS and MCS scores of the acetaminophen and NSAIDs groups at 2 months in a previous study of patients with chronic low back pain [33]. Despite these findings, further studies are needed to determine the differences between the improvement effects of acetaminophen and NSAIDs on QOL.

The incidence of adverse events in the present study is consistent with that reported in previous studies [[34], [35], [36], [37], [38], [39]]. Few grade 3 or higher adverse events were observed within the eight-week study period, demonstrating that acetaminophen and NSAIDs can be administered relatively safely, even in patients 65 years or older. The most common adverse events were gastrointestinal disorders, including dyspepsia, gastric ulcer, abdominal pain, and gastroesophageal reflux disease, which accounted for most adverse event-related treatment discontinuations in the NSAIDs group. The loxoprofen subgroup showed higher GSRS scores than both the acetaminophen group and the celecoxib subgroup. However, unlike pain intensity scales, no universally accepted MCID has been established for the GSRS. Therefore, the clinical relevance of changes in GSRS scores in this study should be interpreted in the context of relative differences between treatment groups, together with the observed rates of gastrointestinal adverse events and treatment discontinuation. Nevertheless, NSAIDs are well known to be associated with gastrointestinal disorders [[34], [35], [36], [37]]. In the present study, the loxoprofen subgroup showed a more pronounced increase in GSRS scores at weeks 2 and 4 than the acetaminophen group. This finding may reflect the early onset of NSAID-related gastrointestinal symptoms and suggests that caution is warranted when prescribing NSAIDs, even for treatment periods of four weeks or shorter. Moreover, because some patients discontinued treatment owing to gastrointestinal disorders, close monitoring is necessary when NSAIDs are used for pain management in older adults.

In the present study, eGFR showed contrasting trends between the acetaminophen and NSAIDs groups. EGFR tended to decrease over time in both NSAID subgroups, including loxoprofen and celecoxib, compared with the acetaminophen group. Although selective COX-2 inhibitors are theoretically considered to have milder renal effects than traditional NSAIDs, previous clinical studies in older adults have suggested that selective COX-2 inhibitors and traditional NSAIDs may affect renal function similarly [38,39]. Consistent with these findings, our results suggest that celecoxib may influence renal function in older patients with osteoarthritis in a manner comparable to loxoprofen. In contrast, the acetaminophen group showed no apparent deterioration in renal function during the study period. Acetaminophen is generally considered to have fewer nephrotoxic effects than NSAIDs [[40], [41], [42], [43]], and our findings suggest that it may have a smaller impact on renal function in older adults with osteoarthritis. However, because the present study was not designed to evaluate changes in CKD stage, it remains unclear whether the observed eGFR changes represent clinically meaningful renal deterioration. Given the increased risk of age-related decline in kidney function among older adults [44], renal function should be monitored when NSAIDs are used for pain management, and acetaminophen may be preferable when renal safety is a concern.

Notably, increased blood pressure was more pronounced in the loxoprofen subgroup than in the acetaminophen group and the celecoxib subgroup. NSAIDs are associated with an increased risk of cardiovascular disorders [45]; therefore, close monitoring and management of adverse effects, such as increased blood pressure, is essential when using NSAIDs in older adults with cardiovascular complications or hypertension.

This study also has some limitations. First, we did not determine differences in the efficacy and safety of the study medications compared with placebo; therefore, we evaluated the validity of the results for each treatment group with respect to MCID values. Second, as this study was conducted within the scope of routine clinical practice, gastrointestinal disorders were assessed using the GSRS questionnaire rather than endoscopic examination. Consequently, a detailed confirmation of gastrointestinal bleeding was not performed, which may have led to an underestimation of the incidence of gastrointestinal disorders. Third, we did not conduct follow-up investigations to confirm whether the increase in alanine transaminase levels caused by acetaminophen was temporary. Fourth, we limited the duration of treatment to a maximum of eight weeks. This study was conducted exclusively in older adults, a population in whom prolonged analgesic administration raises particular safety concerns. Accordingly, the treatment period was designed to confirm analgesic efficacy beyond four weeks while avoiding unnecessary long-term exposure. However, as analgesics may be administered for extended periods in real-world practice, further studies are required to evaluate the efficacy and safety of long-term administration of the study medications. Finally, although differences in pain responses between hip and knee osteoarthritis are recognized, the present study was not designed or powered to conduct joint-specific analyses.

## 5 Conclusion

This study demonstrated that acetaminophen did not meet the prespecified non-inferiority criterion relative to NSAIDs for reducing osteoarthritis-related pain in older adults over an eight-week treatment period, although both treatment groups achieved pain reductions exceeding the MCID. Acetaminophen was associated with a lower incidence of gastrointestinal adverse events and smaller short-term changes in eGFR than NSAIDs. These findings suggest that analgesic selection in older adults with osteoarthritis should be individualized, with careful consideration of both analgesic efficacy and safety as part of a comprehensive management strategy.

## Patient consent for publication

Patients and the public were not involved in the design, conduct, reporting, or dissemination plans of this study.

## Ethics

All patients receive verbal and written information and provide their written informed consent before enrolment. This study is conducted in accordance with the ethical principles stipulated in the “Declaration of Helsinki” (revised October 2013) and “Clinical Trials Act” (announced April 14, 2017, enacted April 1, 2018) established by Japan's Ministry of Health, Labour, and Welfare. This study was approved by the Kyushu University Hospital CRB on January 28, 2021 (KD2020004).

## Author contributions

ME, SZ, TMi, MS, and YN conceived the trial. All the authors were involved in the design of the trial. ME, SZ, TMi, SS, MS, and YN wrote the first draft of the manuscript. All the authors contributed to the revision of the manuscript and approved the final version submitted for publication.

## Data availability statement

The data underlying this manuscript can be obtained upon reasonable request from the corresponding author.

## Role of the funding source

The RETHINK study was funded by AYUMI Pharmaceutical Corporation (Tokyo, Japan) and partly supported by research funds from the Graduate School of Medical Sciences, Kyushu University (Fukuoka, Japan). The sponsor had no control over data collection, data analysis, the interpretation of the study findings, or the writing or publication of this paper. The study data were accessible to the academic investigators, and statistical analyses were performed by an academic statistician independent of the sponsor.

## Declaration of competing interest

The authors declare the following financial interests/personal relationships which may be considered as potential competing interests:

Yasuharu Nakashima reports financial support, article publishing charges, and equipment, drugs, or supplies were provided by Ayumi Pharmaceutical Corporation. Makoto Endo reports a relationship with Daiichi Sankyo Inc that includes: funding grants. Makoto Endo reports a relationship with Ayumi Pharmaceutical Corporation that includes: speaking and lecture fees. Sadamoto Zenda reports a relationship with Maruho Co Ltd that includes: funding grants. Sadamoto Zenda reports a relationship with Merck & Co Inc that includes: funding grants. Sadamoto Zenda reports a relationship with Bristol-Myers Squibb Company that includes: funding grants. Sadamoto Zenda reports a relationship with Ono Pharmaceutical Co Ltd that includes: funding grants. Sadamoto Zenda reports a relationship with Ayumi Pharmaceutical Corporation that includes: consulting or advisory and speaking and lecture fees. Tempai Miyaji reports a relationship with Meaningful Outcome Consulting, Inc. that includes: board membership. Tempai Miyaji reports a relationship with Ayumi Pharmaceutical Corporation that includes: consulting or advisory. Sanae Sakamoto reports a relationship with Ayumi Pharmaceutical Corporation that includes: consulting or advisory. Mototsugu Shimokawa reports a relationship with Ayumi Pharmaceutical Corporation that includes: consulting or advisory. Toshio Takano reports a relationship with Ayumi Pharmaceutical Corporation that includes: employment. Masumi Miyake reports a relationship with Ayumi Pharmaceutical Corporation that includes: employment. Hiroyuki Aono reports a relationship with Ayumi Pharmaceutical Corporation that includes: employment. Yasuharu Nakashima reports a relationship with Daiichi Sankyo Inc that includes: funding grants. Yasuharu Nakashima reports a relationship with Pfizer Inc that includes: funding grants. Yasuharu Nakashima reports a relationship with Ayumi Pharmaceutical Corporation that includes: speaking and lecture fees. If there are other authors, they declare that they have no known competing financial interests or personal relationships that could have appeared to influence the work reported in this paper.
