# Supplementary material for: Comparison of the efficacy and safety of acetaminophen versus NSAIDs for the treatment of chronic pain in older adults with osteoarthritis of the hip and knee: Findings from the randomized, double-blind, parallel-group, non-inferiority RETHINK study
Source: Osteoarthr Cartil Open. 2026 Jul 6;8(3):100850. doi: 10.1016/j.ocarto.2026.100850 (PMC13425900; doi:10.1016/j.ocarto.2026.100850)
Supplement: Multimedia component 1 [file mmc1.pdf]

Comparison of the efficacy and safety of acetaminophen versus NSAIDs for the treatment of chronic pain in older patients with osteoarthritis of the hip and knee: Findings from the randomised, double-blind, parallel-group, non-inferiority RETHINK study

Makoto Endo, Tsutomu Kawano, Masami Tokunaga, Shinya Kawahara, Taro Mawatari, Toshihiko Hara, Yasutaka Tashiro, Masahiro Matsuda, Taishi Sato, Shoji Baba, Akihiko Hamasaki, Toshio Takano, Masumi Miyake, Hiroyuki Aono, Sanae Sakamoto, Tempei Miyaji, Mototsugu Shimokawa, Sadamoto Zenda, Yasuharu Nakashima, on behalf of the RETHINK study group

Supplementary Material

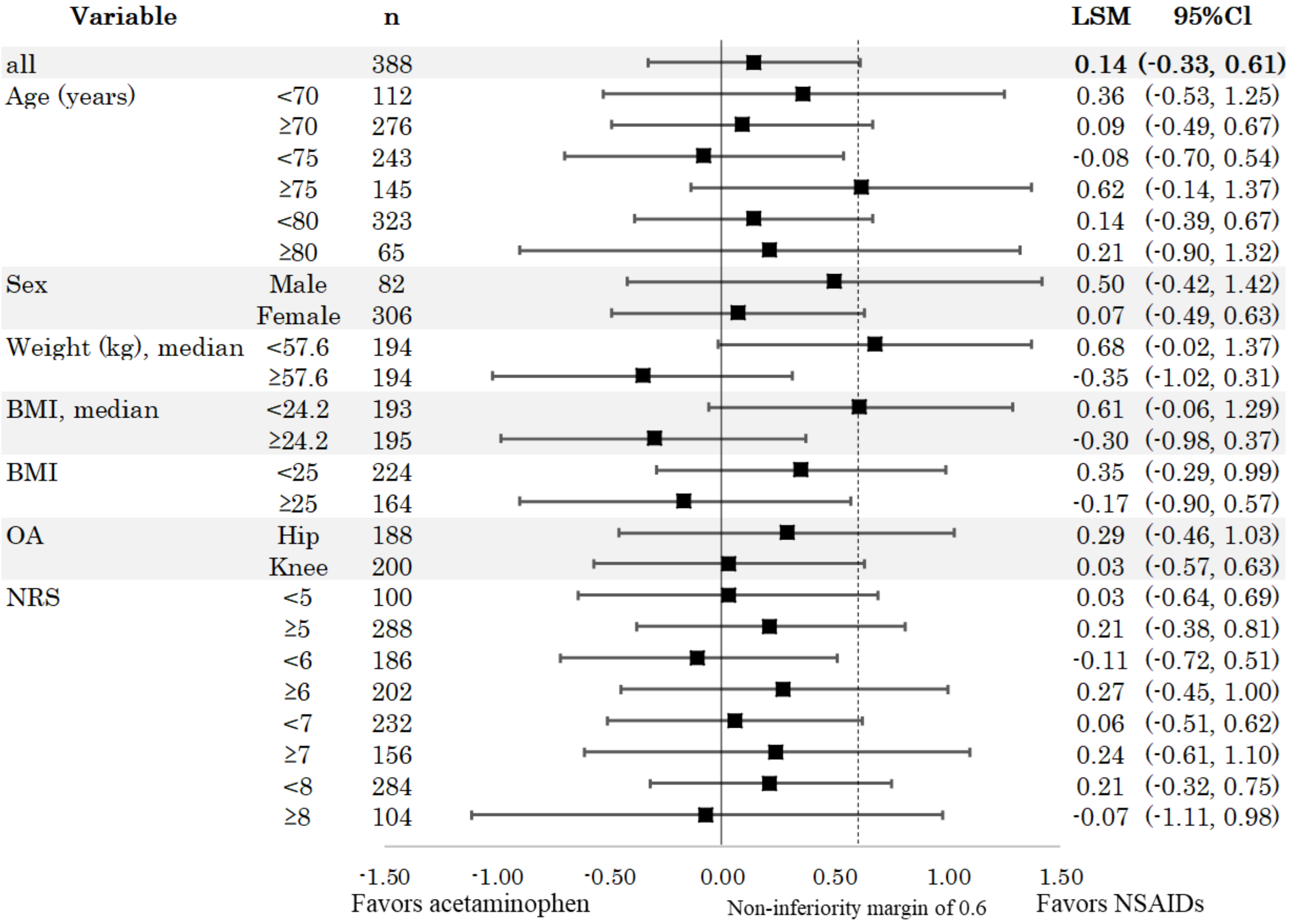

Supplementary Figure S1. Differences in pain score changes from baseline, measured using the numerical rating scale, were analysed in the subgroup analysis

This figure illustrates the difference in least squares mean (LSM) change from baseline between the acetaminophen and the nonsteroidal anti-inflammatory drugs (NSAIDs), as analysed in the subgroup analysis. The primary endpoint for the non-inferiority analysis was the week 8 results (highlighted). The non-inferiority margin was 0.6. The results for week 4 are provided as supplementary information:

Squares, LSM; vertical lines, 95% confidence interval (CI).

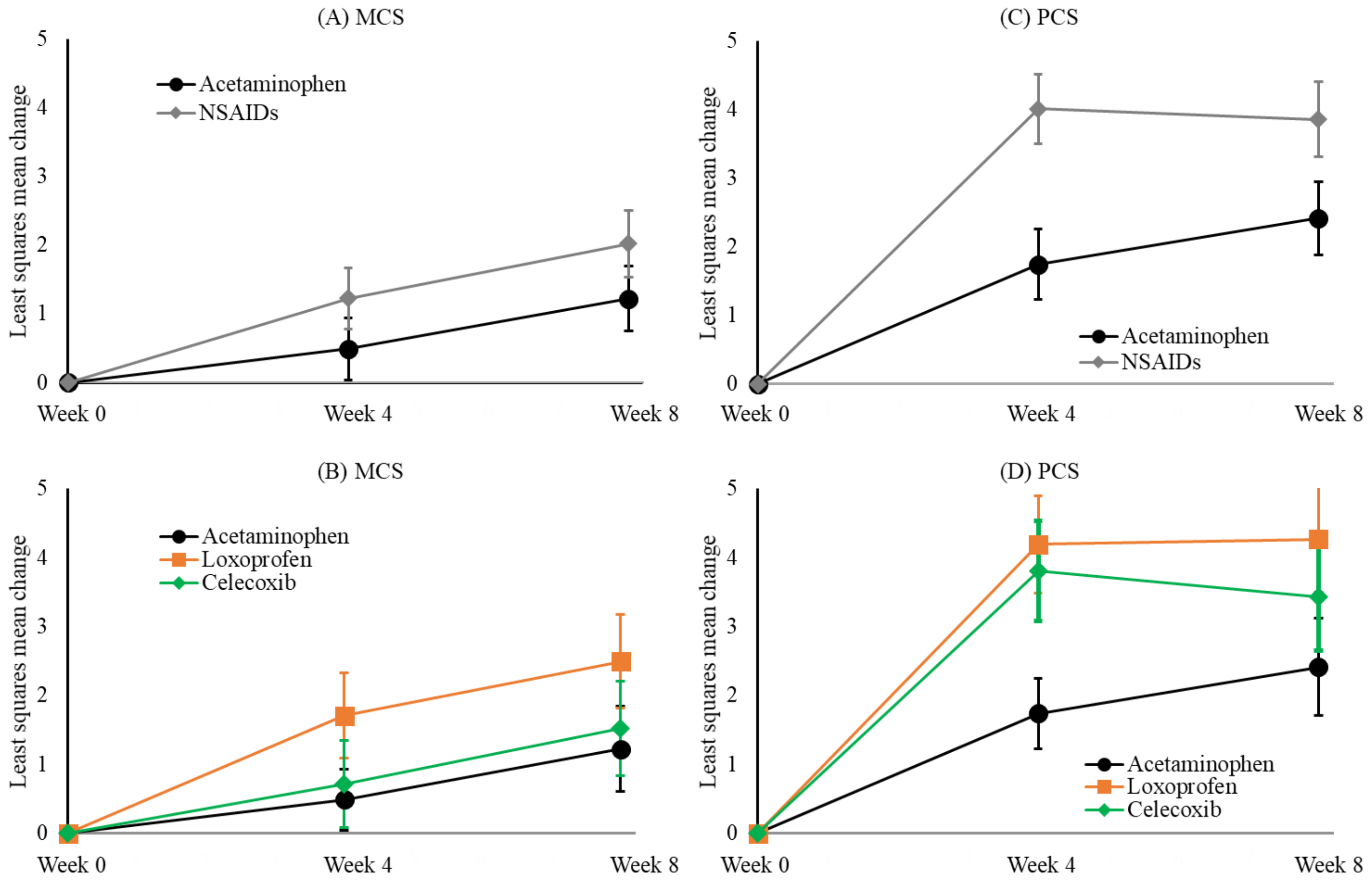

Supplementary Figure S2. Changes in Short Form-8 scores from baseline

This figure illustrates the least squares mean change in the Mental Component Summary (MCS) for (A) the acetaminophen and the nonsteroidal anti-inflammatory drugs (NSAIDs), and (B) the acetaminophen, the loxoprofen, and the celecoxib. It also shows the least squares mean change in the Physical Component Summary (PCS) for (C) the acetaminophen and the nonsteroidal anti-inflammatory drugs (NSAIDs), and (D) the acetaminophen, the loxoprofen, and the celecoxib.

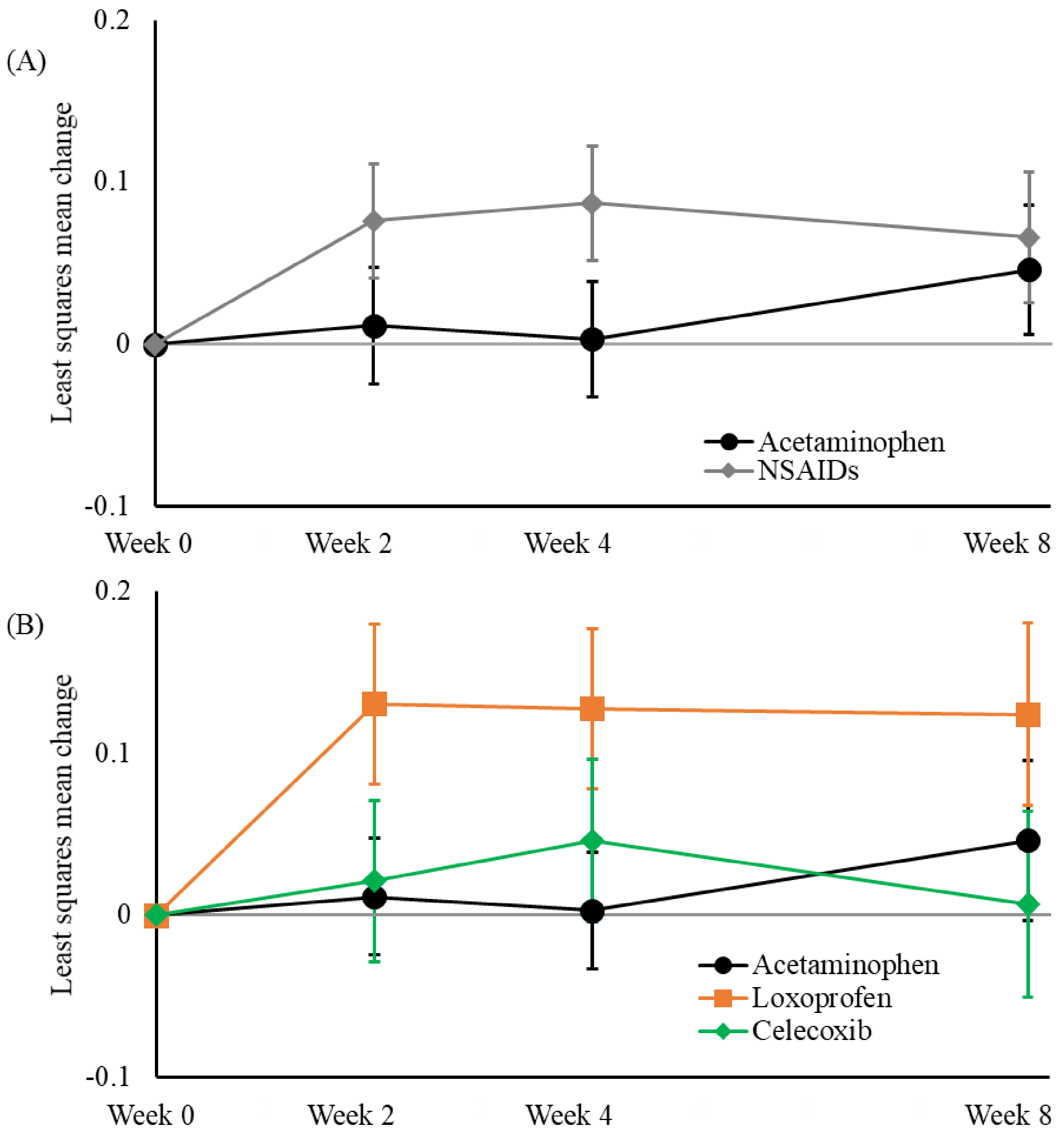

Supplementary Figure S3. Changes in Gastrointestinal Symptom Rating Scale scores from baseline

This figure illustrates the least squares mean change in (A) the acetaminophen and the nonsteroidal anti-inflammatory drugs (NSAIDs), and (B) the acetaminophen, the loxoprofen, and the celecoxib.

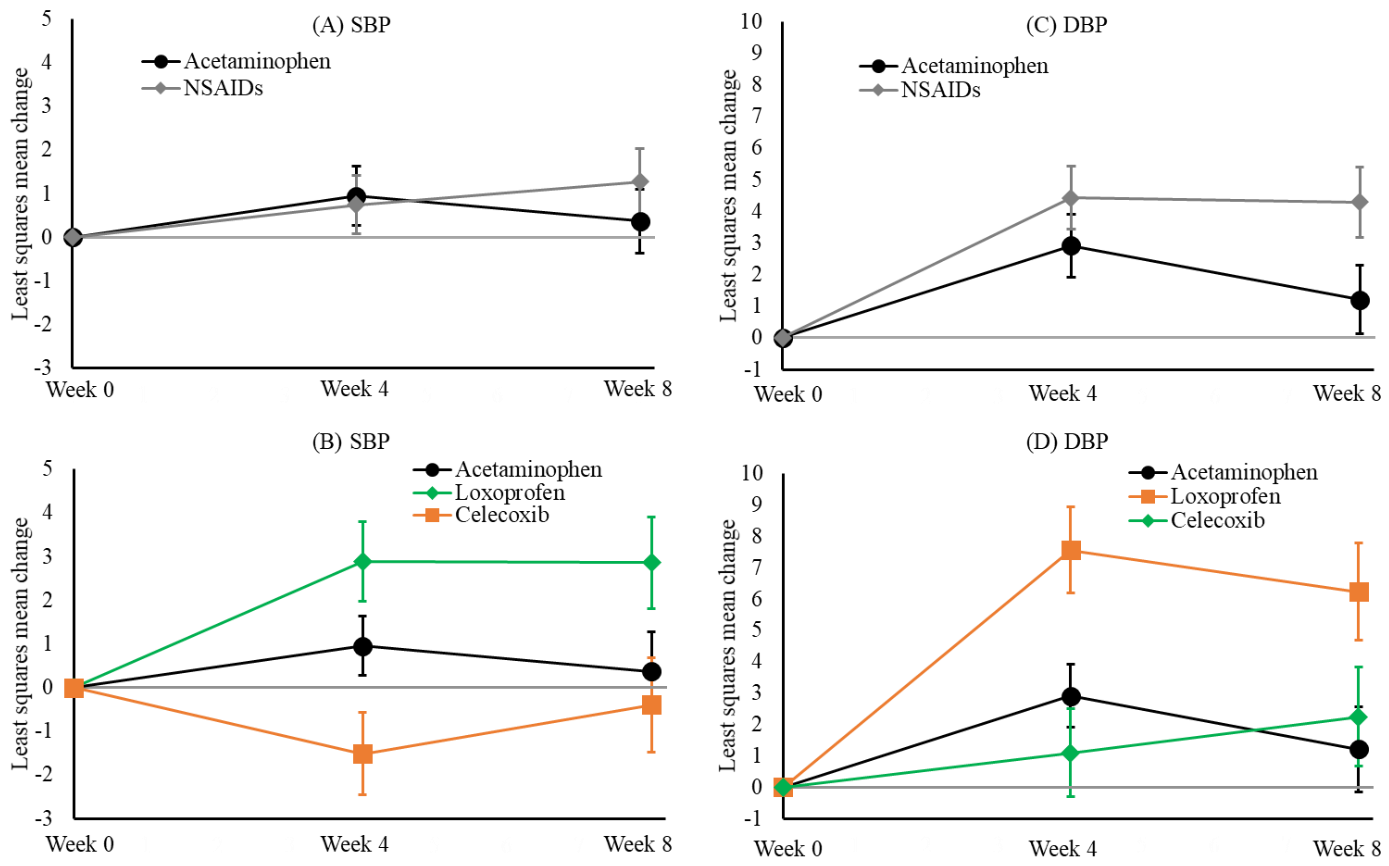

Supplemental Figure S4. Changes in blood pressure values from baseline

This figure illustrates the least squares mean change in systolic blood pressure (SBP) for (A) the acetaminophen and the nonsteroidal anti-inflammatory drugs (NSAIDs), and (B) the acetaminophen, the loxoprofen, and the celecoxib. It also shows the least squares mean change in diastolic blood pressure (DBP) for (C) the acetaminophen and the nonsteroidal anti-inflammatory drugs (NSAIDs), and (D) the acetaminophen, the loxoprofen, and the celecoxib.

Supplemental Table S1. Incidence of adverse events

System/Organ category	Adverse event, n (%)	Acetaminophen (N=193)				Loxoprofen (N=98)				Celecoxib (N=99)			
		Grade 1	Grade 2	Grade 3	all	Grade 1	Grade 2	Grade 3	all	Grade 1	Grade 2	Grade 3	all
Blood and lymphatic system disorders	Anemia	0 (0.0)	0 (0.0)	0 (0.0)	0 (0.0)	1 (1.0)	0 (0.0)	1 (1.0)	2 (2.0)	0 (0.0)	0 (0.0)	0 (0.0)	0 (0.0)
Cardiac disorders	Heart failure	1 (0.5)	0 (0.0)	0 (0.0)	1 (0.5)	0 (0.0)	0 (0.0)	0 (0.0)	0 (0.0)	0 (0.0)	0 (0.0)	0 (0.0)	0 (0.0)
	Palpitations	1 (0.5)	0 (0.0)	0 (0.0)	1 (0.5)	0 (0.0)	0 (0.0)	0 (0.0)	0 (0.0)	0 (0.0)	0 (0.0)	0 (0.0)	0 (0.0)
Ear and labyrinth disorders	Vertigo	1 (0.5)	0 (0.0)	0 (0.0)	1 (0.5)	1 (1.0)	0 (0.0)	0 (0.0)	1 (1.0)	0 (0.0)	0 (0.0)	0 (0.0)	0 (0.0)
Gastrointestinal disorders	Nausea	0 (0.0)	1 (0.5)	0 (0.0)	1 (0.5)	0 (0.0)	0 (0.0)	0 (0.0)	0 (0.0)	1 (1.0)	1 (1.0)	0 (0.0)	2 (2.0)
	Gastritis	0 (0.0)	0 (0.0)	0 (0.0)	0 (0.0)	1 (1.0)	1 (1.0)	0 (0.0)	2 (2.0)	0 (0.0)	1 (1.0)	0 (0.0)	1 (1.0)
	Gastroesophageal reflux disease	1 (0.5)	2 (1.0)	0 (0.0)	3 (1.6)	1 (1.0)	0 (0.0)	0 (0.0)	1 (1.0)	0 (0.0)	0 (0.0)	0 (0.0)	0 (0.0)
	Gastrointestinal disorders – Other	1 (0.5)	0 (0.0)	0 (0.0)	1 (0.5)	0 (0.0)	0 (0.0)	0 (0.0)	0 (0.0)	0 (0.0)	0 (0.0)	0 (0.0)	0 (0.0)
	Gastrointestinal disorders - Other, stomach discomfort	0 (0.0)	0 (0.0)	0 (0.0)	0 (0.0)	1 (1.0)	0 (0.0)	0 (0.0)	1 (1.0)	0 (0.0)	0 (0.0)	0 (0.0)	0 (0.0)
	Gastrointestinal disorders - Other, black stool	0 (0.0)	0 (0.0)	0 (0.0)	0 (0.0)	1 (1.0)	0 (0.0)	0 (0.0)	1 (1.0)	0 (0.0)	0 (0.0)	0 (0.0)	0 (0.0)
	Stomach pain	1 (0.5)	0 (0.0)	0 (0.0)	1 (0.5)	2 (2.0)	1 (1.0)	0 (0.0)	3 (3.1)	2 (2.0)	3 (3.0)	0 (0.0)	5 (5.1)
	Gastric ulcer	3 (1.6)	1 (0.5)	0 (0.0)	4 (2.1)	0 (0.0)	2 (2.0)	0 (0.0)	2 (2.0)	2 (2.0)	3 (3.0)	0 (0.0)	5 (5.1)
	Gastrointestinal disorders - Other, stomach discomfort	0 (0.0)	0 (0.0)	0 (0.0)	0 (0.0)	1 (1.0)	0 (0.0)	0 (0.0)	1 (1.0)	0 (0.0)	0 (0.0)	0 (0.0)	0 (0.0)
	Diarrhea	1 (0.5)	0 (0.0)	0 (0.0)	1 (0.5)	2 (2.0)	0 (0.0)	0 (0.0)	2 (2.0)	1 (1.0)	0 (0.0)	0 (0.0)	1 (1.0)
	Mucositis oral	0 (0.0)	0 (0.0)	0 (0.0)	0 (0.0)	1 (1.0)	1 (1.0)	0 (0.0)	2 (2.0)	0 (0.0)	1 (1.0)	0 (0.0)	1 (1.0)
	Duodenal ulcer	0 (0.0)	0 (0.0)	0 (0.0)	0 (0.0)	0 (0.0)	1 (1.0)	0 (0.0)	1 (1.0)	0 (0.0)	0 (0.0)	0 (0.0)	0 (0.0)
	Dyspepsia	13 (6.7)	4 (2.1)	0 (0.0)	17 (8.8)	8 (8.2)	4 (4.1)	0 (0.0)	12 (12.2)	3 (3.0)	3 (3.0)	0 (0.0)	6 (6.1)
	Abdominal pain	0 (0.0)	0 (0.0)	0 (0.0)	0 (0.0)	2 (2.0)	0 (0.0)	0 (0.0)	2 (2.0)	0 (0.0)	0 (0.0)	0 (0.0)	0 (0.0)
	Constipation	2 (1.0)	0 (0.0)	0 (0.0)	2 (1.0)	1 (1.0)	0 (0.0)	0 (0.0)	1 (1.0)	3 (3.0)	0 (0.0)	0 (0.0)	3 (3.0)
	Vomiting	1 (0.5)	1 (0.5)	0 (0.0)	2 (1.0)	0 (0.0)	0 (0.0)	0 (0.0)	0 (0.0)	0 (0.0)	1 (1.0)	0 (0.0)	1 (1.0)
	Abdominal distension	1 (0.5)	1 (0.5)	0 (0.0)	2 (1.0)	0 (0.0)	0 (0.0)	0 (0.0)	0 (0.0)	1 (1.0)	0 (0.0)	0 (0.0)	1 (1.0)
General disorders and administration site conditions	Malaise	1 (0.5)	0 (0.0)	0 (0.0)	1 (0.5)	0 (0.0)	0 (0.0)	0 (0.0)	0 (0.0)	1 (1.0)	0 (0.0)	0 (0.0)	1 (1.0)
	Edema limbs	0 (0.0)	0 (0.0)	0 (0.0)	0 (0.0)	1 (1.0)	0 (0.0)	0 (0.0)	1 (1.0)	1 (1.0)	0 (0.0)	0 (0.0)	1 (1.0)
Hepatobiliary disorders	Hepatobiliary disorders - Other	1 (0.5)	0 (0.0)	0 (0.0)	1 (0.5)	0 (0.0)	0 (0.0)	0 (0.0)	0 (0.0)	0 (0.0)	0 (0.0)	0 (0.0)	0 (0.0)
Infections and infestations	Infective rhinitis	0 (0.0)	1 (0.5)	0 (0.0)	1 (0.5)	0 (0.0)	0 (0.0)	0 (0.0)	0 (0.0)	0 (0.0)	0 (0.0)	0 (0.0)	0 (0.0)
	Upper respiratory infection	0 (0.0)	0 (0.0)	0 (0.0)	0 (0.0)	1 (1.0)	0 (0.0)	0 (0.0)	1 (1.0)	0 (0.0)	0 (0.0)	0 (0.0)	0 (0.0)
	Shingles	0 (0.0)	1 (0.5)	0 (0.0)	1 (0.5)	0 (0.0)	0 (0.0)	0 (0.0)	0 (0.0)	0 (0.0)	0 (0.0)	0 (0.0)	0 (0.0)
	Urinary tract infection	0 (0.0)	0 (0.0)	1 (0.5)	1 (0.5)	0 (0.0)	1 (1.0)	0 (0.0)	1 (1.0)	0 (0.0)	0 (0.0)	0 (0.0)	0 (0.0)
	Bladder infection	0 (0.0)	1 (0.5)	0 (0.0)	1 (0.5)	0 (0.0)	0 (0.0)	0 (0.0)	0 (0.0)	0 (0.0)	0 (0.0)	0 (0.0)	0 (0.0)
	Infections and infestations - Other, COVID-19	1 (0.5)	1 (0.5)	0 (0.0)	2 (1.0)	1 (1.0)	1 (1.0)	0 (0.0)	2 (2.0)	1 (1.0)	0 (0.0)	0 (0.0)	1 (1.0)
Injury, poisoning and procedural complications	Injury, poisoning and procedural complications - Other, chest wall contusion	0 (0.0)	1 (0.5)	0 (0.0)	1 (0.5)	0 (0.0)	0 (0.0)	0 (0.0)	0 (0.0)	0 (0.0)	0 (0.0)	0 (0.0)	0 (0.0)
	Fracture	0 (0.0)	0 (0.0)	0 (0.0)	0 (0.0)	0 (0.0)	0 (0.0)	0 (0.0)	0 (0.0)	0 (0.0)	1 (1.0)	0 (0.0)	1 (1.0)
	Spinal fracture	0 (0.0)	0 (0.0)	1 (0.5)	1 (0.5)	0 (0.0)	0 (0.0)	0 (0.0)	0 (0.0)	0 (0.0)	0 (0.0)	0 (0.0)	0 (0.0)
	Wrist fracture	0 (0.0)	0 (0.0)	1 (0.5)	1 (0.5)	0 (0.0)	0 (0.0)	0 (0.0)	0 (0.0)	0 (0.0)	0 (0.0)	0 (0.0)	0 (0.0)
	Wound dehiscence	0 (0.0)	1 (0.5)	0 (0.0)	1 (0.5)	0 (0.0)	0 (0.0)	0 (0.0)	0 (0.0)	0 (0.0)	0 (0.0)	0 (0.0)	0 (0.0)
Investigations	Increase in CPK level	1 (0.5)	0 (0.0)	0 (0.0)	1 (0.5)	0 (0.0)	0 (0.0)	0 (0.0)	0 (0.0)	0 (0.0)	0 (0.0)	0 (0.0)	0 (0.0)
	Increase in creatinine level	1 (0.5)	0 (0.0)	0 (0.0)	1 (0.5)	0 (0.0)	0 (0.0)	0 (0.0)	0 (0.0)	0 (0.0)	0 (0.0)	0 (0.0)	0 (0.0)
	Weight gain	0 (0.0)	0 (0.0)	0 (0.0)	0 (0.0)	0 (0.0)	1 (1.0)	0 (0.0)	1 (1.0)	0 (0.0)	0 (0.0)	0 (0.0)	0 (0.0)
	Increase in GGT level	4 (2.1)	1 (0.5)	0 (0.0)	5 (2.6)	0 (0.0)	0 (0.0)	0 (0.0)	0 (0.0)	0 (0.0)	0 (0.0)	0 (0.0)	0 (0.0)
Metabolism and nutrition disorders	Hyperkalemia	0 (0.0)	0 (0.0)	0 (0.0)	0 (0.0)	0 (0.0)	0 (0.0)	0 (0.0)	0 (0.0)	2 (2.0)	0 (0.0)	0 (0.0)	2 (2.0)
Nervous system disorders	Somnolence	0 (0.0)	0 (0.0)	0 (0.0)	0 (0.0)	1 (1.0)	0 (0.0)	0 (0.0)	1 (1.0)	0 (0.0)	0 (0.0)	0 (0.0)	0 (0.0)

	Headache	1 (0.5)	0 (0.0)	0 (0.0)	1 (0.5)	0 (0.0)	1 (1.0)	0 (0.0)	1 (1.0)	0 (0.0)	0 (0.0)	0 (0.0)	0 (0.0)
	Dizziness	2 (1.0)	0 (0.0)	0 (0.0)	2 (1.0)	0 (0.0)	0 (0.0)	0 (0.0)	0 (0.0)	0 (0.0)	0 (0.0)	0 (0.0)	0 (0.0)
Renal and urinary disorders	Renal and urinary disorders - Other	0 (0.0)	0 (0.0)	0 (0.0)	0 (0.0)	0 (0.0)	0 (0.0)	0 (0.0)	0 (0.0)	1 (1.0)	0 (0.0)	0 (0.0)	1 (1.0)
	Cystitis noninfective	0 (0.0)	0 (0.0)	0 (0.0)	0 (0.0)	1 (1.0)	0 (0.0)	0 (0.0)	1 (1.0)	0 (0.0)	0 (0.0)	0 (0.0)	0 (0.0)
	Urinary frequency	0 (0.0)	0 (0.0)	0 (0.0)	0 (0.0)	1 (1.0)	0 (0.0)	0 (0.0)	1 (1.0)	0 (0.0)	0 (0.0)	0 (0.0)	0 (0.0)
	Non-infective cystitis	0 (0.0)	0 (0.0)	0 (0.0)	0 (0.0)	0 (0.0)	0 (0.0)	0 (0.0)	0 (0.0)	0 (0.0)	1 (1.0)	0 (0.0)	1 (1.0)
Respiratory, thoracic and mediastinal disorders	Respiratory, thoracic and mediastinal disorders - Other, hemoptysis	0 (0.0)	0 (0.0)	0 (0.0)	0 (0.0)	1 (1.0)	0 (0.0)	0 (0.0)	1 (1.0)	0 (0.0)	0 (0.0)	0 (0.0)	0 (0.0)
Skin and subcutaneous tissue disorders	Rash acneiform	0 (0.0)	1 (0.5)	0 (0.0)	1 (0.5)	0 (0.0)	0 (0.0)	0 (0.0)	0 (0.0)	0 (0.0)	0 (0.0)	0 (0.0)	0 (0.0)
	Pruritus	0 (0.0)	0 (0.0)	0 (0.0)	0 (0.0)	0 (0.0)	0 (0.0)	0 (0.0)	0 (0.0)	1 (1.0)	0 (0.0)	0 (0.0)	1 (1.0)
	Bullous dermatitis	1 (0.5)	0 (0.0)	0 (0.0)	1 (0.5)	0 (0.0)	0 (0.0)	0 (0.0)	0 (0.0)	0 (0.0)	0 (0.0)	0 (0.0)	0 (0.0)
	Skin and subcutaneous tissue disorders - Other, rash	0 (0.0)	1 (0.5)	0 (0.0)	1 (0.5)	0 (0.0)	0 (0.0)	0 (0.0)	0 (0.0)	0 (0.0)	0 (0.0)	0 (0.0)	0 (0.0)
	Eczema	1 (0.5)	0 (0.0)	0 (0.0)	1 (0.5)	0 (0.0)	0 (0.0)	0 (0.0)	0 (0.0)	0 (0.0)	1 (1.0)	0 (0.0)	1 (1.0)
Vascular disorders	Hematoma	0 (0.0)	0 (0.0)	0 (0.0)	0 (0.0)	0 (0.0)	0 (0.0)	0 (0.0)	0 (0.0)	1 (1.0)	0 (0.0)	0 (0.0)	1 (1.0)
	Hypertension	0 (0.0)	0 (0.0)	0 (0.0)	0 (0.0)	0 (0.0)	0 (0.0)	0 (0.0)	0 (0.0)	0 (0.0)	1 (1.0)	0 (0.0)	1 (1.0)

Supplemental Table S2. Number and percentage of patients with hepatobiliary function levels that exceeded the upper limit of normal

	n (%)	Acetaminophen (N=183)	NSAIDs		
			All (N=187)	Loxoprofen (N=96)	Celecoxib (N=91)
Week 4	AST	14 (7.7)	4 (2.1)	1 (1.0)	3 (3.3)
	ALT	30 (16.4)	9 (4.8)	4 (4.2)	5 (5.5)
	GTP	44 (24.0)	7 (3.7)	4 (4.2)	3 (3.3)
	ALP	11 (6.0)	5 (2.7)	3 (3.1)	2 (2.2)
	T-bil	5 (2.7)	4 (2.1)	2 (2.1)	2 (2.2)
Week 8	AST	12 (6.7)	9 (5.4)	6 (7.1)	3 (3.7)
	ALT	27 (15.2)	7 (4.2)	5 (5.9)	2 (2.4)
	GTP	42 (23.6)	3 (1.8)	2 (2.4)	1 (1.2)
	ALP	7 (3.9)	5 (3.0)	3 (3.5)	2 (2.4)
	T-bil	5 (2.8)	3 (1.8)	2 (2.4)	1 (1.2)

NSAIDs, nonsteroidal anti-inflammatory drugs; AST, aspartate aminotransferase; ALT, alanine aminotransferase; GTP, gamma-glutamyl transpeptidase; ALP, alkaline phosphatase; T-bil, total bilirubin.
